# Metabolomic Analysis of Follicular Fluid in Normal-Weight Patients with Polycystic Ovary Syndrome

**DOI:** 10.3390/biomedicines12081810

**Published:** 2024-08-09

**Authors:** Jiayue Yu, Yiqiu Wei, Zhourui Zhang, Jiao Chen, Rongrong Fu, Peng Ye, Suming Chen, Jing Yang

**Affiliations:** 1 Reproductive Medicine Center, Renmin Hospital of Wuhan University, Wuhan 430060, China; 18883289206@163.com (J.Y.); weiyiqiu@whu.edu.cn (Y.W.); drchenjiao@whu.edu.cn (J.C.); 2 The Institute for Advanced Studies, Wuhan University, Wuhan 430072, China; 18794831171@163.com (Z.Z.); 13895224152@163.com (R.F.); 3 Department of Pharmacy, Renmin Hospital of Wuhan University, Wuhan 430060, China; yp800111@163.com

**Keywords:** polycystic ovary syndrome, follicular fluid, metabolomics, LC–MS, reproductive

## Abstract

Background: This study aimed to examine the differential variations in the metabolic composition of follicular fluid (FF) among normal-weight patients with polycystic ovary syndrome (PCOS) and controls and to identify potential biomarkers that may offer insights into the early identification and management of these patients. Methods: We collected FF samples from 45 normal-weight women with PCOS and 36 normal-weight controls without PCOS who were undergoing in vitro fertilization–embryo transfer. An untargeted metabolomic study of collected FF from infertile women was performed using high-performance liquid chromatography–tandem spectrometry (LC-MS). The tendency of the two groups to separate was demonstrated through multivariate analysis. Univariate analysis and variable importance in projection were used to screen out differential metabolites. Metabolic pathway analysis was conducted using the Kyoto Encyclopedia of Genes and Genomes (KEGG), and a diagnostic model was established using the random forest algorithm. Results: The metabolomics analysis revealed an increase in the expression of 23 metabolites and a decrease in that of 10 metabolites in the FF of normal-weight women with PCOS. According to the KEGG pathway analysis, these differential metabolites primarily participated in the metabolism of glycerophospholipids and the biosynthesis of steroid hormones. Based on the biomarker combination of the top 10 metabolites, the area under the curve value was 0.805. The concentrations of prostaglandin E2 in the FF of individuals with PCOS exhibited an inverse association with the proportion of high-quality embryos (*p* < 0.05). Conclusions: Our research identified a distinct metabolic profile of the FF from normal-weight women with PCOS. The results offer a broader comprehension of the pathogenesis and advancement of PCOS, and the detected differential metabolites could be potential biomarkers and targets for the treatment of PCOS.

## 1. Introduction

Polycystic ovary syndrome (PCOS) is a prevalent metabolic and reproductive disease affecting fertile women and has a global incidence rate of 8–13% [1]; the primary clinical features include hyperandrogenemia (HA), insulin resistance (IR), menstrual disorders, and follicular dysplasia [2]. The diagnostic criteria for PCOS are still nonuniform, and the Rotterdam diagnostic criteria are the most widely used [3]. The pathogenesis and molecular basis of PCOS remain unclear and are related to genetic and environmental factors and lifestyle, among others [4]. Although assisted reproductive technology has become a commonly used treatment method for ovulatory dysfunction infertility in PCOS because of the abnormal follicular development caused by endocrine disorders and metabolic disorders, poor oocyte quality is often seen during treatment [5]. Patients with PCOS also have a significantly higher risk of suffering from cardiovascular disease, endometrial cancer, hepatic steatosis, and type 2 diabetes (T2DM) than the normal population [6]. Despite extensive research on PCOS, the cause is still unclear, and PCOS remains a challenge in the medical management of infertility.

The follicular fluid (FF) mainly contains the blood passing through the blood follicle barrier and the secretions produced by cells inside the follicle, particularly the granulosa cells (GCs). The FF serves as an intricate microenvironment for the development of oocytes, maturation of follicles, and interactions between germ and somatic cells [7]. The primary constituents of FF include growth factors, cytokines, amino acids, proteins, lipids, and polysaccharides. The levels of multiple substances in FF, such as 25-hydroxyvitamin D [8], fatty acids [5], C-type natriuretic peptide [9], and noncoding RNAs [10], are associated with oocyte quality and pregnancy outcome. The lack of high-quality oocytes in patients with PCOS may be due to abnormal FF and localized autocrine–paracrine regulatory mechanisms in the ovary. Specific biochemical characteristics of the FF surrounding oocytes can affect oocyte quality, fertilization potential, and embryonic development more than those of other biological samples. The FF is collected when women undergo IVF–embryo transfer (IVF-ET), and as a noninvasive matrix, it has emerged as a crucial resource for investigating PCOS.

Metabolomics refers to the qualitative and quantitative analysis of small-molecule metabolites in specific conditions and times, thereby discovering the corresponding response of organisms to changes in internal and external environments and subsequent overall metabolic changes. Metabolites are downstream products of multiple intracellular factors involved in gene expression, RNA transcription, protein transposon activity, and enzyme function [11]. Therefore, metabolomics can provide a more real-time assessment of the actual state of the organism than other omics. PCOS is usually accompanied by varying degrees of metabolic disorders involving multiple metabolic pathways, and can especially include metabolic disorders of amino acids, carbohydrates, and lipids. Metabolomics has been shown to play a crucial role in comprehending the disease status in PCOS and uncovering novel biomarkers to optimize the pregnancy outcome of patients [12].

Obesity affects 50–80% of patients with PCOS [13]; nevertheless, with PCOS exhibiting a wide range of clinical and biochemical symptoms, a significant number of individuals with PCOS have normal weight. Compared with obese women with PCOS, normal-weight women with PCOS have lower rates of IR and metabolic syndrome but more severe HA [14]. The levels of lysophosphatidylcholine (LPC) vary among PCOS patients with varying body weights. The normal-weight PCOS group has been shown to have elevated levels of LPC (18:2), LPC (26:0), LPC (20:4), and LPC (18:1) in comparison with the overweight and obese PCOS group [15]. This suggests that body weight affects the metabolism in PCOS and that the pathogenesis in normal-weight patients with PCOS may differ from that in patients with PCOS who have obesity. Most current studies have focused on the overall metabolism of PCOS but have not examined metabolites in the FF of normal-weight women with PCOS. In this research, we employed metabolomics using liquid chromatography–tandem mass spectrometry (LC–MS) to gain a comprehensive understanding of the metabolic alterations in FF among normal-weight patients with PCOS.

## 2. Materials and Methods

### 2.1. Subjects

A prospective clinical trial including 81 infertile patients with IVF-ET was conducted between December 2020 and October 2022 in the Reproductive Medicine Center at Renmin Hospital of Wuhan University. Permission was given by the Ethics Committee of the Renmin Hospital of Wuhan University (WDRY2019-K077), and all participants signed informed consent. Patients were classified into normal-weight women with PCOS (PCOS, *n* = 45) and without PCOS (CON, *n* = 36). PCOS individuals were recruited based on the Rotterdam criteria [3]. The ovarian function of individuals in the non-PCOS category was within the normal range. The inclusion criteria were as follows: (1) aged between 22 and 35 years; (2) body mass index (BMI) from 18.5 to 24 kg/m^2^ [16]; (3) undergoing the first IVF-ET cycle. The exclusion criteria were as follows: (1) use of immunomodulatory, cytokine, cytotoxic, or hormonal drugs three months before treatment; (2) endometriosis; (3) history of ovarian surgery and pelvic tuberculosis; (4) immune insufficiency pregnancy; (5) unexplained infertility; (6) other endocrine diseases present simultaneously.

### 2.2. Follicular Fluid Collection

An individualized ovarian stimulation program was used based on the ovarian reserve of each patient. In our experimental center, the commonly used protocols for patients with PCOS are the gonadotropin-releasing hormone (GnRH) antagonist and ultralong GnRH agonist protocols. The GnRH antagonist protocol was as follows: Patients began intramuscular injection of human menopausal gonadotropin (Gn) and follicle-stimulating hormone (FSH) on the second day of their menstrual cycle, and when the average follicle diameter was ≥14 mm and the estradiol (E2) level was >734–1101 pmol/L, subcutaneous injections of GnRH antagonist at 0.25 mg/d, vaginal ultrasound, and sex hormone examination were performed every 2–3 d, and the dosage adjusted according to follicular development. If the patient met the downregulation criteria, the GnRH antagonist was changed to 0.05 mg/d until the trigger day. The ultralong GnRH agonist protocol was as follows: Patients began intramuscular injection of long-acting GnRH agonist 3.75 mg on the second day of their menstrual cycle. After 28–35 d, ultrasound and endocrine examination were performed; if the standard of downregulation was reached, Gn treatment was initiated, which would have been the same as the GnRH antagonist protocol. When the diameters of two follicles were >18 mm or those of three follicles were >17 mm, an intramuscular injection of human chorionic gonadotropin (HCG) 8000–10,000 U was used to induce ovulation. Oocytes were retrieved by puncture under vaginal ultrasound guidance approximately 34–36 h following the HCG injection. The removed oocytes were cultured, and the FF without blood contamination was transferred to Eppendorf tubes. Then, FF was centrifuged at 1500 rpm for 10 min at 4 °C to remove cell debris and insoluble particles. Approximately 1.5 mL of supernatant was collected and stored at −80 °C for subsequent testing.

### 2.3. Fertilization and Embryo Quality Assessment

Semen was processed by density gradient centrifugation, and short IVF fertilization was performed 3–4 h after oocyte retrieval. The fertilization outcome was determined based on whether pronuclei appeared after 16–18 h of sperm addition or whether pronuclei were not found but cleavage had occurred. Clinically, observing two prokaryotic nuclei and two polar bodies was defined as regular fertilization. Embryos were categorized into grades I–IV based on the size, morphology, and fragmentation rate of the cleavage sphere; embryos grade III and above were defined as transferable embryos, and grade I and II embryos were classified as high-quality embryos.

### 2.4. Sample Preparation

FF samples were thawed at 4 °C and vortexed for 40 s, and 100 µL of each sample was added to a fresh tube. A total of 400 µL volume of precooled methanol was added, vortexed for 2 min, and then centrifuged at 15,000 rpm for 10 min at 4 °C. And 400 µL of the supernatant was taken and frozen at −20 °C for 2 h. Next, the sample was centrifuged at 15,000 rpm for 10 min at 4 °C, and 300 µL of supernatant was blown dry with nitrogen and then stored at −80 °C. Before injection, the sample was reconstituted with 100 μL of 80% pre-cooled methanol aqueous solution, vortexed for 1 min, and stored at −20 °C for 2 h. Then, the sample was centrifuged at 15,000 rpm for 10 min at 4 °C. Subsequently, 70 μL of the supernatant was used for LC–MS analysis, and 10 μL from each sample was mixed thoroughly as the quality control (QC) sample for assessing the stability of the LC-MS analysis.

### 2.5. LC-MS Analysis and Data Acquisition

LC–MS analysis was performed using a timsTOF Pro mass spectrometer (Bruker Daltonics, Bremen, Germany) coupled with an Ultimate 3000 liquid system (Thermo Scientific, Waltham, MA, USA). To improve the accuracy and range of the identification, two complementary liquid chromatogram methods were applied, which were hydrophilic interaction liquid chromatography (HILIC) and reversed-phase liquid chromatography (RPLC). The HILIC method was based on a previous study [17]. As for the RPLC method, a Waters C18 column (2.1 mm × 100 mm, 1.7 µm) was utilized. The mobile phase A was H_2_O with 0.1% formic acid, and the mobile phase B was ACN with 0.1% formic acid. A gradient elution was used as follows: 0–2 min, 2% B; 2–17 min, 2–100% B; 17–20 min, 100% B; 20–21.1 min, 100–2% B; 21.1–21 min, 2% B. The temperature of the column oven was maintained at 50 °C, and the injector plate was maintained at 4 °C. The injection volume was 5 µL. The specific parameters were as follows: scanning range: 20–1300 Da; collision energy: 20 eV; dry gas flow rate: 10.0 L/min; injection gas: 2.2 bar; capillary voltage: 3.6 kV; and capillary temperature: 220 °C. The data-dependent acquisition method was utilized with the top 3 ions selected. Before the analysis, the mass spectrometer was externally calibrated with sodium formate and equilibrated with ten injections of QC samples. During the data acquisition, the FF samples were randomly injected, with QC samples inserted every ten samples to ensure instrument stability.

### 2.6. Statistical Analysis

Raw data were preprocessed using Metaboscape 4.0, with steps including peak extraction, alignment, and noise removal. Then, peak annotation was applied by matching the exact molecular weight and MS/MS spectrum with the HMDB database (http://www.hmdb.ca/, accessed on 9 March 2023) and an in-house database. Then, the peak area data was treated in MetaboAnalyst 5.0 (www.metaboanalyst.ca). First, the total ion current normalization was employed to exclude the effects of multiple external factors. Then, features with a relative standard deviation > 25% in QC samples were filtered out. The rest of the features were brought into SIMCA-P 14.0 (Umetrics, Umeaa, Switzerland) for principal component analysis (PCA) and orthogonal partial least squares discriminant analysis (OPLS-DA) with the missing values filled in with 1/5 of the smallest positive value. The OPLS-DA model was modeled with 200 permutations to evaluate the reproducibility and the risk of overfitting. To evaluate the quality of the model, R^2^X (the rate of explanation for the X model), R^2^Y (the rate of explanation for the distinction between groups), and Q2 (the model’s predictive capability) were combined as the model output parameters. R^2^X > 0.5 indicated that the model quality was good, and the closer the values of R^2^Y and Q2 were to 1, the better the model effect was. In addition, variable importance in projection (VIP) values of each metabolite depending on the OPLS-DA model were exported. Besides the multivariate analysis, a univariate analysis was also used to screen differential metabolites. The fold change (FC) value of each metabolite was calculated on the basis of the average value in the two groups, and the p value was calculated by the two-sided unpaired Wilcox test. Metabolites with VIP > 1, FC > 1.2, and *p* value < 0.05 were defined as significantly differential metabolites. Pathway enrichment analysis was performed using Metaboanalyst 5.0. Spearman’s correlation coefficients between differential metabolites and ovarian function indices were obtained using the psych package (v.2.3.6) in R (v.4.2.3). Correlation networks of differential metabolites were mapped by Cytoscape (v.3.9.1). Then, the normal-weight PCOS group was distinguished from the control group using a diagnostic model constructed by the random forest algorithm. Furthermore, the baseline characteristics of the subjects were compared in SPSS software (version 25.0, SPSS Inc., Chicago, IL, USA).

## 3. Results

### 3.1. Clinical Characteristics

The clinical characteristics of patients are listed in Table 1. The normal-weight PCOS and control groups were well-matched for age and BMI. There were no significant differences in FBG level, years of infertility, concentration of bE2 and progesterone, days of stimulation, and dosages of Gn between the two groups. The bLH, LH/FSH, AMH, bAFC were significantly higher, but bFSH was significantly lower in normal-weight PCOS patients. In an analysis of the oocytes and fertilization results, there were no significant differences in the number of 2PN fertilizations, 2PN cleavages, high-quality embryos on the third day, and the rates of MII oocytes between the two groups. However, the number of oocytes retrieved and MII oocytes were significantly higher and the high-quality embryo rate was lower in the normal-weight PCOS group.

### 3.2. Multivariate Analysis of Metabolites

**Figure 1:**
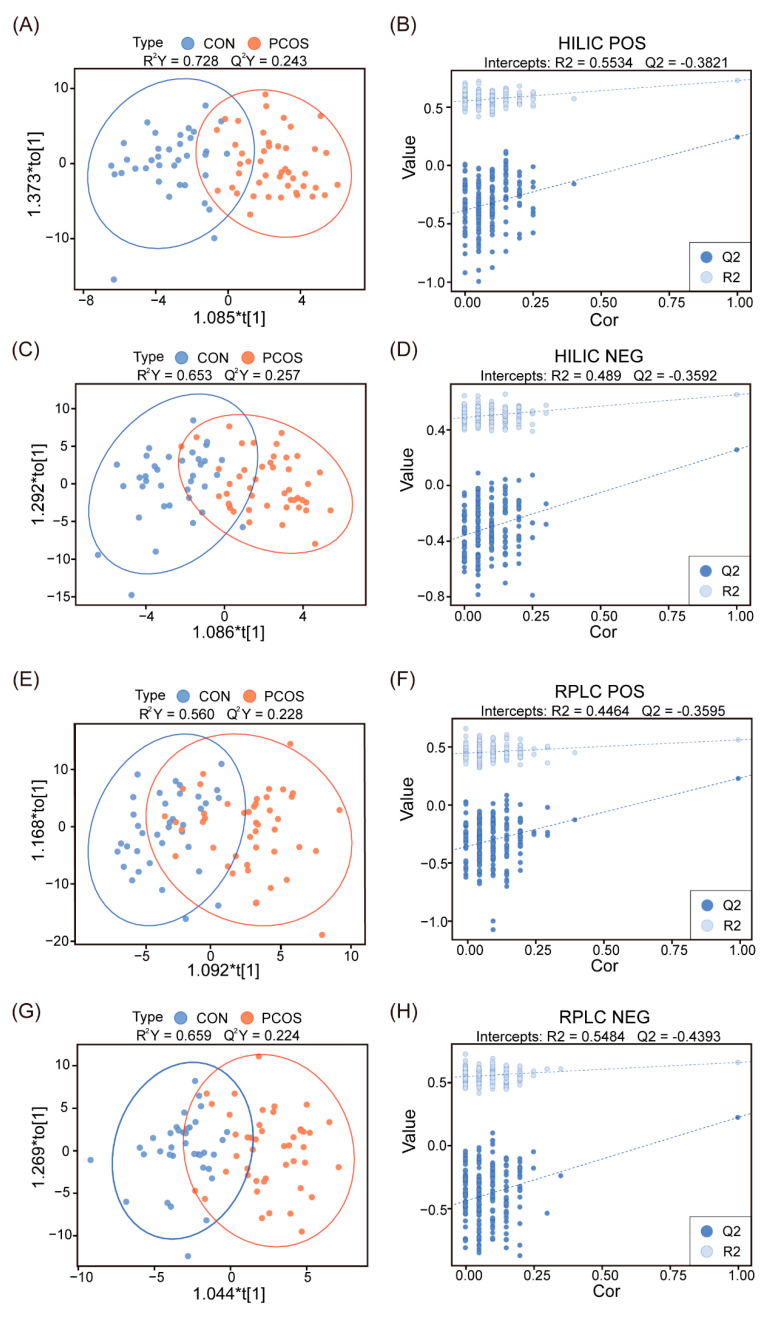
Separation trends between the normal-weight PCOS group (red circles) and the control group (blue circles) were described using OPLS-DA score scatter plots with permutation tests. (**A**) OPLS-DA score scatter plot of positive ion mode in HILIC; (**B**) permutation test of positive ion mode in HILIC; (**C**,**D**) negative ion mode in HILIC; (**E**,**F**) positive ion mode in RPLC; (**G**,**H**) negative ion mode in RPLC.

Both liquid chromatogram methods, RPLC and HILIC, were used to detect metabolites in the FF of the normal-weight PCOS and the normal-weight control groups. The PCA and OPLS-DA models were constructed to reveal the overall difference in metabolomics between the two groups. The OPLS-DA model showed that the two groups were distributed in different regions (Figure 1A,C,E,G). The value of R^2^Y was significantly more than 0.5, suggesting that the model had a relatively good explanatory rate. Q^2^Y represents the model’s predictive ability in the Y matrix, and Q^2^Y values > 0.2 are acceptable in consideration of the complexity and variability of clinical samples. The permutation test was used to effectively assess overfit (Figure 1B,D,F,H), and it showed the values of Q2 and R2 in the four models. The intersections of the regression line of the Q2 point and the ordinate are less than 0 in the four models, indicating that these models were not overfitted. In addition, the PCA model did not show significant separation between the two groups (Figure S1), suggesting the difference in metabolomics was slight. During data acquisition, the QC samples were inserted to assess the stability of continuous injection. The QC samples were closely clustered with correlation coefficients > 0.97, indicating the stability and reproducibility of the metabolomics data (Figure S1).

### 3.3. Analysis of Differential Metabolites

**Figure 2:**
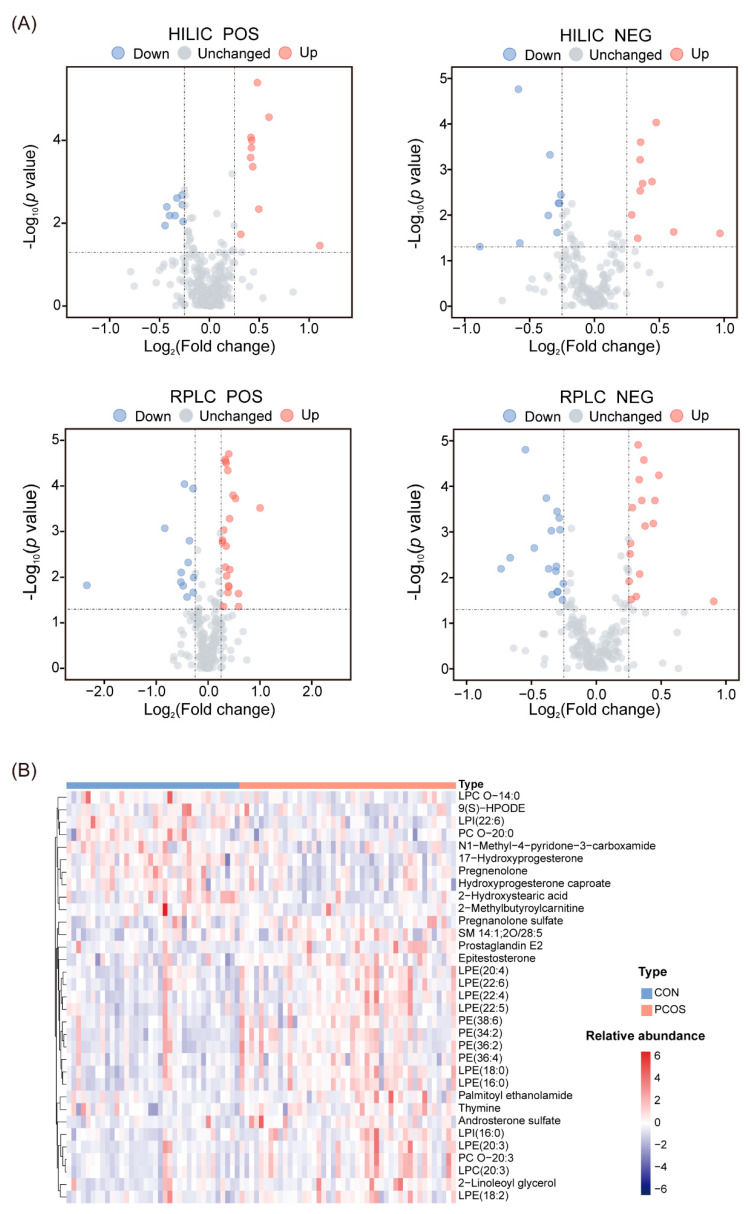
Identification of differential metabolites between the two groups. (**A**) Volcano plots show differential metabolites in different modes, where red dots represent upregulated metabolites and blue dots represent downregulated metabolites; (**B**) hierarchical clustering heatmap of differential metabolites.

After peak extraction, alignment, and annotation, a total of 7046 features were found in this study, of which 694 had annotations. The univariate analysis result of all features with annotation showed that 58 features were upregulated and 39 were downregulated in the normal-weight PCOS group (Figure 2A). Combined with the VIP value > 1 from the OPLS-DA model, there were finally 23 upregulated and 10 downregulated metabolites. The heatmap of differential metabolites (Figure 2B) showed apparent differences between the two groups, mainly manifested by the upregulation of multiple lipids in the normal-weight PCOS group.

### 3.4. Pathway Enrichment Analysis and Correlation Analysis of Differential Metabolites

**Figure 3:**
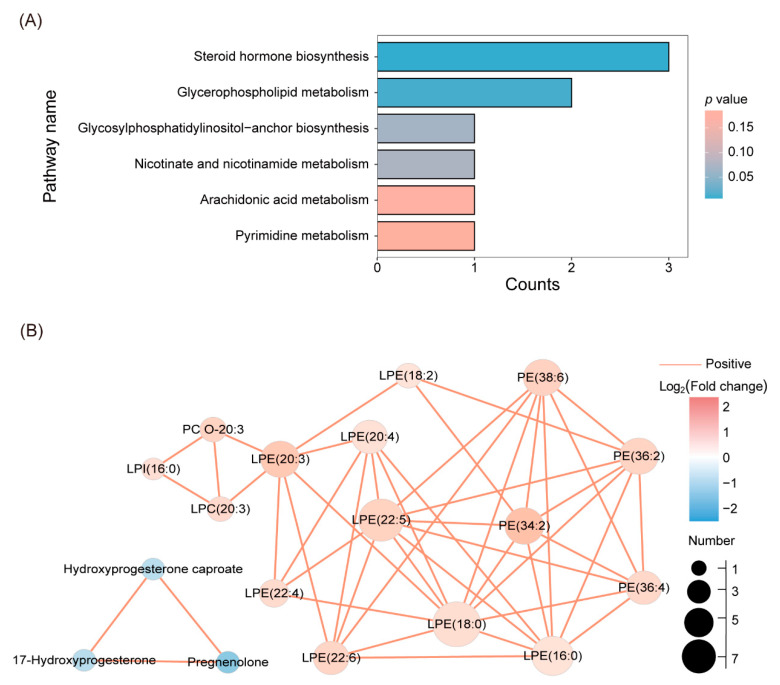
(**A**) KEGG pathway enrichment analysis of differential metabolites. The blue represents the *p* value, which means the metabolic pathway was significantly influenced. The size of the point represents the number of relevant metabolites involved in this pathway. (**B**) Correlation network diagram of differential metabolites based on *r* > 0.6 (or *r* < −0.6) and *p* value < 0.05.

To explore the overall metabolic alterations during the development of PCOS, metabolic pathway analysis of all 33 differential metabolites was performed. Based on the KEGG database, there were six enriched metabolic pathways, mainly in steroid hormone biosynthesis (*p* = 0.0073) and glycerophospholipid metabolism (*p* = 0.0135) (Figure 3A). In addition, the correlation analysis was performed, and positive correlations were found for several metabolites (Figure 3B and Figure S2), indicating a similar variation tendency.

### 3.5. Analysis of Clinical Indicator Correlations

**Figure 4:**
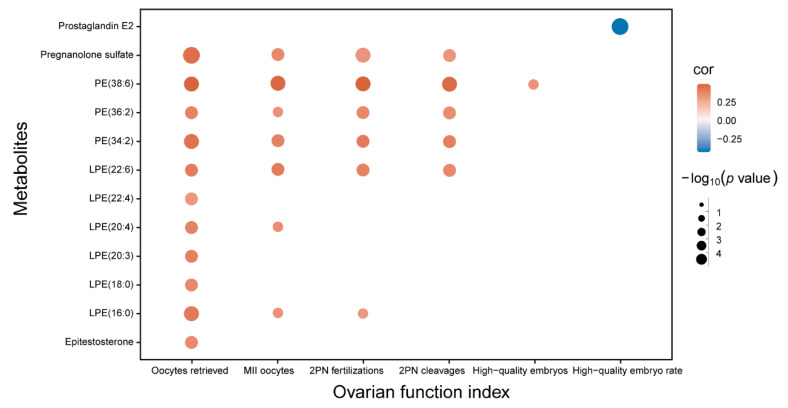
Spearman correlation coefficients of clinical indicators and differential metabolites. The correlation coefficient (*r*) value is shown by the color of the point on the map while the *p* value is indicated by its size.

Different types of correlation were present between the differential metabolites and clinical indicators. Most of the differential lipids were positively correlated with these clinical indicators, whereas prostaglandin E2 (PGE2) was negatively correlated with the rate of high-quality embryos (Figure 4).

### 3.6. Establishment of a Diagnostic Model Based on Metabolites

**Figure 5:**
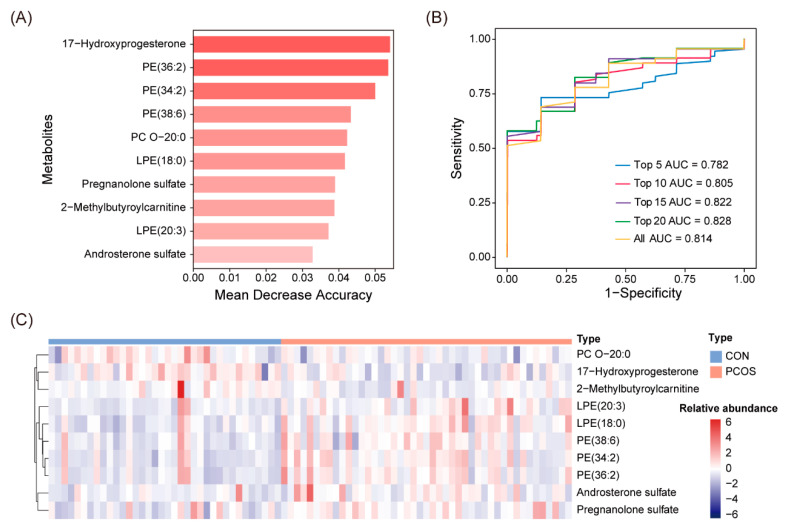
Development and testing of a diagnostic model based on 10 metabolites. (**A**) The ranking of classification importance of the top 10 metabolites. (**B**) The ROC curve and AUC value of the combined diagnostic model of metabolites; the ROC curves for the top 5, 10, 15, 20, and all metabolites are presented individually by blue, red, green, purple, and yellow curves. (**C**) Heatmap of the top 10 differential metabolites.

The random forest algorithm was used to construct a diagnostic model to distinguish normal-weight patients with PCOS or without PCOS. Firstly, the differential metabolites were ranked in order of importance for classification depending on the value of mean decrease accuracy (Figure 5A). Then, the top 5, 10, 15, and 20 differential metabolites were selected to build models. The area under the curve (AUC) value of the model containing the top 10 metabolites (AUC = 0.805) was just slightly inferior to that of all metabolites (AUC = 0.814) (Figure 5B). Therefore, the top 10 metabolites were selected to construct a diagnostic panel in consideration of efficiency and convenience (Figure 5C and Figure S3).

## 4. Discussion

The FF is the microenvironment where oocytes develop, and the metabolic composition of the FF can reflect the quality of oocytes to some extent. Herein, we used an untargeted metabolomics method to examine FF samples from 81 subjects. Comparing the FF of normal-weight women with PCOS to that of normal-weight women without PCOS, we discovered 33 differential metabolites. Most of these metabolites were enriched in steroid hormone biosynthesis, glycerophospholipid metabolism, glycosylphosphatidylinositol-anchored biosynthesis, nicotinate and nicotinamide metabolism, arachidonic acid metabolism, and pyrimidine metabolism, which are closely related to oocyte growth and development. In this section, we thoroughly analyze the possible processes of the diverse metabolites.

In recent years, increasing attention has been paid to the metabolic profile of follicular fluid in infertile women to determine whether differential metabolites in follicular fluid are associated with infertility [18,19,20]. Lazzarino et al. [18] used a metabolomics approach to identify differences in metabolites in follicular fluid between control and infertile women and developed a biomarker score based on 27 differential metabolites, which was significantly and negatively correlated with the number of oocytes, mature oocytes, fertilized oocytes, blastocysts, and high-quality blastocysts, suggesting that the level of metabolites in the follicular fluid may reflect the quality of oocytes and influence the developmental ability of embryos. The metabolic profiles of follicular fluid from healthy women and cancer patients undergoing oocyte cryopreservation were significantly different. The metabolic disorders found in the follicular fluid of cancer patients may be related to the poorer quality of oocytes, suggesting that oocytes with metabolic profiles similar to those of healthy patients should be selected for cryopreservation. The metabolomics of follicular fluid is a powerful means of identifying and selecting oocytes for cryopreservation [21]. A nontargeted metabolomics study based on ultrahigh-performance liquid chromatography connected with quadrupole time-of-flight mass spectrometry (UHPLC-MS) evaluated the clinical efficacy of acupoint compresses for the treatment of phlegmatic PCOS patients, and the results showed that the levels of pseudouridine, phenol, 2-oxoadipic acid, 9R,10S-EpOME, DL-lactate, nicotinamide, and DL-indole-3-lactic acid were downregulated after the treatment and clinically manifested as a decrease in the number of days to Gn and an increase in the number of available embryos, suggesting that this treatment is effective in improving clinical symptoms and IVF treatment outcomes in PCOS patients of the phlegm-dampness type [22]. In addition, information on the biochemical composition of FF can help to develop personalized therapies or diets for infertile patients, thereby improving their symptoms and pregnancy outcomes [23]. The above results suggest that follicular fluid metabolomics can be used both to identify potential biomarkers of disease and to predict embryonic developmental potential and apply to disease treatment and efficacy evaluation.

Thymine was detected in the FF of patients with PCOS. Pyrimidine metabolism plays a vital role in the synthesis of RNA and DNA and the formation of proteins. Pyrimidine synthesis is involved in cell proliferation and apoptosis, and the physiological homeostasis of pyrimidines is extremely sensitive to cell proliferation and apoptosis processes [24]. Patients with PCOS usually have decreased cell proliferation and increased apoptosis in GCs, which provide nutrients to oocytes, affecting follicular development [25]. β-Aminoisobutyric acid (BAIBA), the final catabolic metabolite of thymine, significantly reduces blood glucose levels in T2DM and mice with IR [26]. Meanwhile, the human serum BAIBA level is negatively correlated with serum insulin concentration [27]. Studies have reported that the thymine level in FF was notably elevated in the PCOS-IR group compared to that in the PCOS patients without IR (PCOS-NIR) group [28]. These findings imply that thymine may have an impact on PCOS metabolism by regulating insulin levels.

N1-Methyl-4-pyridone-3-carboxamide (4PY) is the final product of nicotinamide adenine dinucleotide (NAD) degradation and is engaged in nicotinamide and nicotinate ester metabolism. Abnormal NAD levels can cause cellular redox potential imbalance, oxidative stress changes, and mitochondrial dysfunction [29]. Although the underlying mechanisms of PCOS remain unclear, inflammation and mitochondrial dysfunction have been reported in the GCs of patients with PCOS. Inflammation can reduce NAD levels in the GCs of these patients, and supplementing nicotinamide riboside can restore NAD levels and alleviate mitochondrial dysfunction in these GCs [30]. Levels of 4PY, as the final result of the metabolism of NAD, are decreased in the FF of patients with PCOS, and this matches up with the findings of the current experimental investigation, implying an insufficient pool of NAD in the follicular cells from patients with PCOS [31]. Knocking down two critical genes in NAD biosynthesis reduced NAD levels in mouse ovaries, impairing oocyte quality and ultimately reducing fertilization capacity and impairing early embryonic development [32]. Thus, 4PY deficiency in patients with PCOS may cause mitochondrial dysfunction and inflammation, which may affect follicle and oocyte development.

Dysregulation of steroid hormone synthesis is usually observed in PCOS. In this study, we found that compared to those in the control group, the levels of various steroid hormones were changed in the FF of normal-weight patients with PCOS, and those of androsterone sulfate (AnS), epitestosterone, and pregnanolone sulfate were upregulated, whereas those of 17-hydroxyprogesterone (17-OHP), pregnenolone, and hydroxyprogesterone caproate were downregulated. Pregnenolone is an endogenous steroid hormone precursor of progesterone, androgens, estrogens, mineralocorticoids, glucocorticoids, and neuroactive steroids [17]. Guo et al. used stable isotope labeling–LC-MS for quantitative analysis to detect steroid hormones in body fluids and found that the content of pregnenolone in the FF of the PCOS group was greatly decreased [33], which is consistent with our results. Mechanisms of androgen overproduction include increased luteinizing hormone secretion, hyperinsulinemia, and increased responsiveness of ovarian theca cells to Gn stimulation [34]. After being stimulated with Gns, PCOS patients had elevated levels of 17-OHP in their blood, according to many studies [35]. However, the level of 17-OHP in PCOS patients has been shown to be unrelated to follicle size [36]. Zhang et al. found that IL-18 increased the secretion of 17-OHP and androstenedione and upregulated the expression of critical steroidogenesis-related genes Cyp11a1 and CyP17A1, which may be the pathway for the mode of action of IL-18 in PCOS and also suggests that 17-OHP contributes to the pathogenesis of PCOS [37]. PCOS has similar clinical features to certain diseases, and diagnosing PCOS remains a difficult task. 17-OHP is the primary steroid used to exclude nonclassical congenital adrenal hyperplasia [38]. 17-OHP combined with other biomarkers also improves the diagnostic efficiency of PCOS [31]. Several studies have shown no significant difference in 17-OHP in the FF from patients with PCOS [33], unlike the results of this investigation. This is possible because the patient’s weight was not considered, and the sample size was limited. Epitestosterone belongs to the group of endogenous steroids, which are testosterone isomers. The biological significance of epitestosterone is controversial, and this is generally considered an inactive metabolite in the organism. However, several studies found that epitestosterone exerts antiandrogenic effects by competing with testosterone for androgen receptor sites [39,40]. Epitestosterone can inhibit cyclooxygenase-mediated arachidonic acid metabolism, but this cannot directly prove that epitestosterone has anti-inflammatory effects [40]. There is insufficient evidence to support the hypothesis that epitestosterone contributes to the development of PCOS. AnS is the most abundant 5-alpha-reduced androgen metabolite in the serum. AnS was shown to be increased in the plasma from patients with PCOS [41], but there was no significant change in the embryo culture medium (ECM) of PCOS patients [42]. AnS was upregulated in FF in the PCOS group and is a reliable indicator of the miscarriage rate [42], suggesting that changes in AnS may affect PCOS pathophysiology and embryonic development.

PGE2, produced by enzymatic metabolism from arachidonic acid, is essential for female reproductive functions [5,43,44]. Previous studies reported that inhibiting the rate-limiting enzyme for PGE2 synthesis in follicles or genetically knocking down the expression of the PGE2 receptor gene can prevent ovulation [45]. During oocyte meiosis, PGE2 can promote the phosphorylation of MAPK, maintain spindle tissue morphology, and indirectly promote oocyte maturation and division [46]. PGE2 may affect embryo implantation by activating the cAMP-signaling pathway by binding to the receptor EP2 [47]. PGE2 also plays a vital role in embryonic development in patients with PCOS, and the level of PGE2 in FF in the PCOS group was increased in several studies [5,44]. In this study, PGE2 was negatively correlated with the rate of high-quality embryos, and other studies also found that PGE2 was positively correlated with the number of MII and 2PN oocytes [5]. ZNF217 is expressed less often in GCs in PCOS patients and induces inflammation through PGE2, and PGE2 inhibits the expression of ZNF217 to establish a feedback loop, which may contribute to PCOS pathogenesis [2]. ATF4 dysfunction in patients with PCOS can inhibit HCG-induced COX2 expression and PGE2 production, affecting ovulation [48]. The pathophysiological mechanism of PGE2 in PCOS patients and the embryonic development process have not been extensively studied and deserve attention.

Phospholipids are structural components of cell and organelle membranes and are involved in many critical pathophysiological cellular processes [49]. Previous research showed that a series of phospholipids in the FF of patients with PCOS changed significantly [50,51], which corresponds with the findings of this investigation. Wang et al. [52] performed a metabolomic analysis of follicular fluid and found that LPE 22:6 mediated the association of cadmium and mercury with the risk of PCOS, suggesting that high exposure to metals may affect the metabolism of glycerophospholipids, which may adversely affect female reproductive function. The upregulation of LPE in PCOS FF may be due to reduced activity of lysophospholipid acyltransferase 4 (LPCAT4) and/or LYPLA1 [51]. FF was centrifuged at 15,000 rpm, retaining some organelles and vesicles. Therefore, the elevated level of PE may not be due to PCOS [50], and a more in-depth study is needed to determine whether PE is involved in the pathological process of PCOS. LPC is closely related to apoptosis [53], inflammation, and glucose regulation [54]. During follicle development, LPC is more abundant in large follicles than in small follicles, indicating that LPC may play a role in the growth of follicles and the maturation of oocytes and may act as a target for therapy to improve human follicles’ and oocytes’ growth and development [55]. LPC in FF has been shown to change significantly with age, affecting the maturation of oocytes and decreasing fertility in older women [56]. BMI and LPC are negatively correlated [55], and serum LPC levels in obese patients with PCOS are lower than those in lean controls [14]. LPC is an essential mediator in fatty-acid-induced IR and is involved in several important activities, including the absorption and utilization of glucose. LPC may function as a stand-alone insulin signal to control blood glucose levels [57]. Thus, obesity and insulin resistance may affect LPC production and, consequently, fertility in patients with PCOS. LPI stimulates insulin secretion by increasing intracellular calcium ion concentration by activating G-protein coupled receptor (GPR) 55 on pancreatic β cells [58]. Higher levels of LPI (18:0) correlate with higher fasting insulin, free-androgen index (FAI), HbA1c, IR and adiposity, and lower sex hormone-binding globulin (SHBG), indicating that LPI has a vital role in PCOS metabolism [59].

Abnormalities were also observed in other metabolites in this study. Palmitoyl ethanolamide (PEA) is an endogenous fatty acid amide and a nuclear transcription factor agonist class member. Peroxisome proliferator-activate receptor-alpha (PPARalpha), a significant target of PEA, regulates the expression of inflammation-related genes when activated [60]. PEA levels rise as a defensive measure to hinder inflammatory pathways, causing tissue harm and functional decline when the inflammatory state lingers [61]. PCOS may be linked to a mild inflammatory condition and inflammatory markers. In this study, PEA was found to be upregulated. Although there are currently no studies showing the role PEA plays in PCOS, it can also be conceivable that PEA levels in FF may be related to inflammation, thereby affecting the development of PCOS. 2-Methylbutyrylcarnitine is an acylcarnitine. Acylcarnitines enable fatty acids to be transported into the mitochondrial matrix for oxidation and thus participate in cellular energy metabolism. Carnitine has antioxidant properties by scavenging ROS, regulating the function of enzymes responsible for ROS production, and safeguarding mitochondrial metabolism [62]. FF from women with PCOS displays heightened levels of ROS and molecular oxidative harm, while decreased overall antioxidant capacity is linked to poor embryo quality [63]. The use of different subclasses of carnitine in an animal model of PCOS could have beneficial effects on the ovarian microenvironment [64]. The expression of 2-methylbutyrylcarnitine in FF in the PCOS group was downregulated, indicating that the total antioxidant capacity of PCOS was reduced. 9(S)-HPODE, a long-chain lipid hydroperoxide family member and a linoleic acid metabolic product, can induce intracellular glutathione oxidation [65]. We discovered in this work that 9(S)-HPODE levels were downregulated in the FF of PCOS patients for the first time, which may indicate new relevant pathways.

Our findings provide new insights into studying PCOS pathological mechanisms and investigating new biomarkers for the diagnosis of PCOS. However, there are some limitations to this research. One is that ovarian stimulation with exogenous gonadotropins may alter FF metabolites, and thus, this may not represent their natural state. Second, the composition of FF is related to the development stage of follicles, and our results only reflect the changes in the metabolic composition of FF before ovulation. Third, this study is cross-sectional and cannot draw a clear cause-and-effect relationship between metabolites and PCOS. Therefore, both in vitro and in vivo studies are required to confirm the presence of variable metabolite levels and understand how these metabolites contribute to PCOS pathogenesis.

## 5. Conclusions

In this study, we comprehensively analyzed metabolic changes in FF of normal-weight patients with PCOS using nontargeted metabolomic technology, focusing on the microenvironment of the oocyte. Diagnostic models based on metabolite changes allowed us to distinguish normal-weight patients with PCOS from normal-weight women without PCOS during IVF. Correlation analyses revealed strong associations between the differential metabolites and clinical indicators, which would be valuable for enhancing the oocyte maturation culture system in vitro. In conclusion, the use of follicular fluid metabolomics to explore the pathogenesis of PCOS and the metabolic pathways of oocytes at the small molecule level can improve the quality of oocytes and embryos and also provide new ideas and a theoretical basis for the clinical development of precise diagnostic and therapeutic strategies for PCOS.

## Figures and Tables

**Table 1:** Clinical characteristics of normal-weight PCOS and control groups.

	PCOS (*n* = 45)	CON (*n* = 36)	*p* Value
Age (year)	30.11 ± 2.41	30.86 ± 2.97	0.213
BMI (kg/m^2^)	21.65 ± 1.55	21.2 ± 1.42	0.186
FBG (mmol/L)	4.9 ± 0.62	4.91 ± 0.39	0.970
Duration of infertility(year)	3.24 ± 2.27	3.28 ± 3.14	0.956
bFSH (mIU/mL)	6.54 ± 1.37	7.62 ± 2.25	0.014 *
bLH (mIU/mL)	5.76 ± 3.43	4.12 ± 2.32	0.013 *
LH/FSH	0.87 ± 0.45	0.54 ± 0.24	<0.001 **
bE2 (pg/mL)	40.07 ± 12.48	36.69 ± 15.12	0.903
P (ng/mL)	0.63 ± 0.35	0.85 ± 0.64	0.074
AMH (ng/mL)	8.36 ± 4.11	3.95 ± 2.16	<0.001 **
bAFC (n)	29.24 ± 9.55	18.33 ± 7.26	<0.001 **
Gn dosage (IU)	1597.56 ± 705.1	1793.14 ± 465.4	0.138
Days of stimulation (days)	10.47 ± 2.51	10 ± 1.94	0.362
Oocytes retrieved (n)	18.13 ± 7.52	13.97 ± 6.98	0.013 *
MII oocytes (n)	12.13 ± 5.83	9.5 ± 4.94	0.034 *
2PN fertilizations (n)	9.51 ± 5.37	7.64 ± 4.37	0.095
2PN cleavages (n)	9.31 ± 5.27	7.56 ± 4.4	0.112
High-quality embryos (n)	4.64 ± 3.38	4.81 ± 3.62	0.837
MII oocytes rate (%)	66.77 ± 3.38	70.06 ± 3.16	0.445
2PN fertilization rate (%)	52.92 ± 3.33	57.01 ± 3.45	0.400
2PN cleavage rate (%)	97.97 ± 0.78	98.93 ± 0.63	0.343
High-quality embryo rate (%)	48.97 ± 3.89	61.87 ± 4.78	0.038 *

Data are expressed as mean ± SD. The Student’s *t*-test or Mann–Whitney test was used to calculate the *p* values depending on the normality of the data. PCOS, polycystic ovary syndrome; CON, normal ovarian reserve; BMI, body mass index; FBG, fasting blood glucose; bFSH, basal follicle-stimulating hormone; bLH, basal luteinizing hormone; bE2, basal estrogen; P, progesterone; AMH, anti-Müllerian hormone; bAFC, basal antral follicle count; Gn, gonadotropin; MII, second meiotic division; 2PN, 2 pronuclei; MII oocyte rate, the ratio of MII number to the obtained oocyte number; 2PN fertilization rate, the ratio of 2PN number to the obtained oocyte number; 2PN cleavage rate, the ratio of 2PN cleavage number to the 2PN fertilization number; high-quality embryo rate, the ratio of high-quality embryo number to the 2PN cleavage number. * *p* < 0.05, ** *p* < 0.001.

## Data Availability

Data will be made available upon reasonable request to the corresponding author.

## Supplementary Materials

The following supporting information can be downloaded at: https://www.mdpi.com/article/10.3390/biomedicines12081810/s1, Table S1: Differential metabolites in FF. Figure S1: PCA analysis and the correlation analysis matrix of all QCs in four modes. Figure S2: Heatmap of correlations between differential metabolites. Figure S3: Histogram of the top 10 metabolites according to the ranking of classification importance. The PCOS group is displayed in red, and the control group is displayed in blue. The y-axis represents the integrated data of peak area normalized by TIC from LC-MS. The *p* values were calculated by a two-sided unpaired Wilcox test. * *p* <0.05, ** *p* <0.01, *** *p* <0.001.
