# Supplementary material for: Metabolomic Analysis of Follicular Fluid in Normal-Weight Patients with Polycystic Ovary Syndrome
Source: Biomedicines. 2024 Aug 9;12(8):1810. doi: 10.3390/biomedicines12081810 (PMC11352029; doi:10.3390/biomedicines12081810)
Supplement: Supplementary file 1 [file biomedicines-12-01810-s001.zip › biomedicines-3132217-supplementary.pdf]

Supplementary Materials

Metabolomic Analysis of Follicular Fluid in Normal-Weight Patients with Polycystic Ovary Syndrome

Jiayue Yu [1,†], Yiqiu Wei [1,†], Zhourui Zhang [2], Jiao Chen [1], Rongrong Fu [2], Peng Ye [3], Suming Chen [2,*] and Jing Yang [1,*]

[1] Reproductive Medicine Center, Renmin Hospital of Wuhan University, Wuhan 430060, China; 18883289206@163.com (J.Y.); weiyiqiu@whu.edu.cn (Y.W.); drchenjiao@whu.edu.cn (J.C.)
[2] The Institute for Advanced Studies, Wuhan University, Wuhan 430072, China; 18794831171@163.com (Z.Z.); 13895224152@163.com (R.F.)
[3] Department of Pharmacy, Renmin Hospital of Wuhan University, Wuhan 430060, China; yp800111@163.com
* Correspondence: sm.chen@whu.edu.cn (S.C.); dryangjing@whu.edu.cn (J.Y.)
† These authors equally contributed to this work.

Supplementary Figures

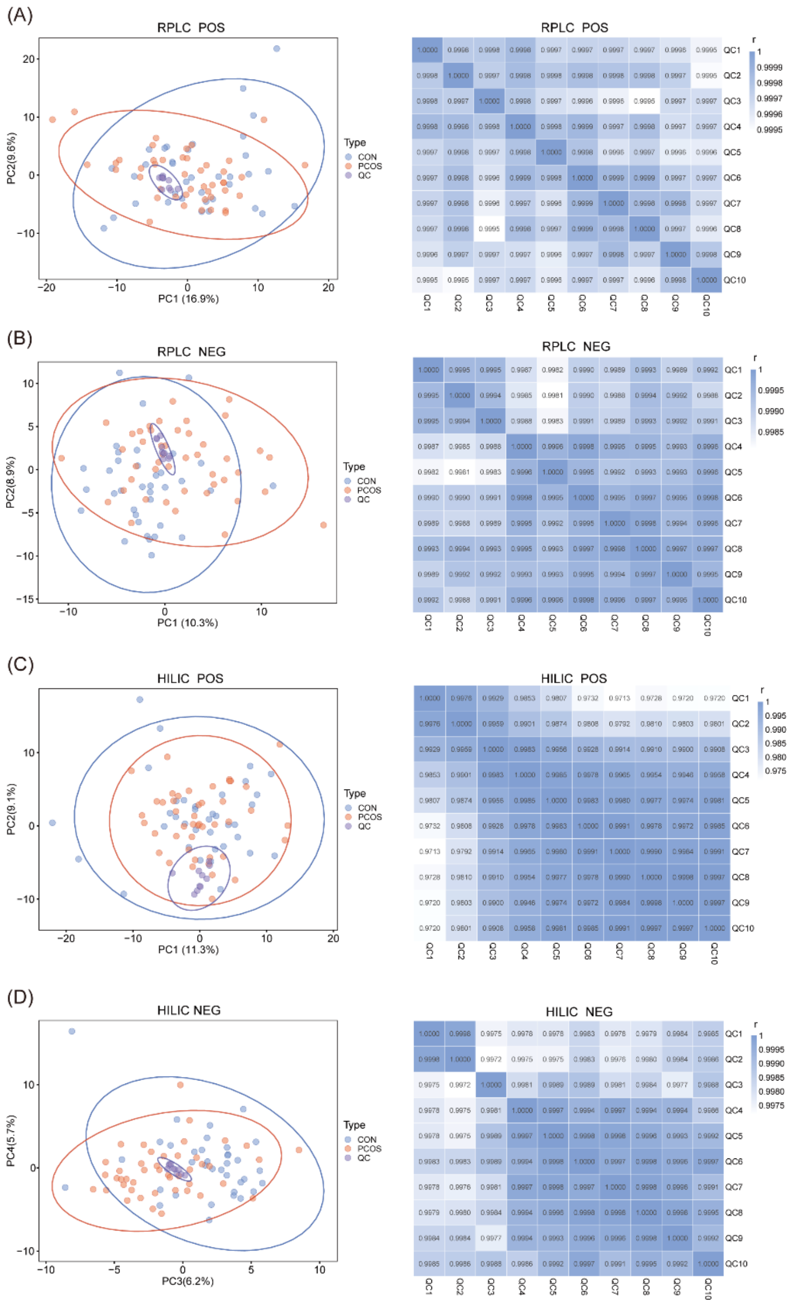

Figure S1. PCA analysis and the correlation analysis matrix of all QCs in four modes.

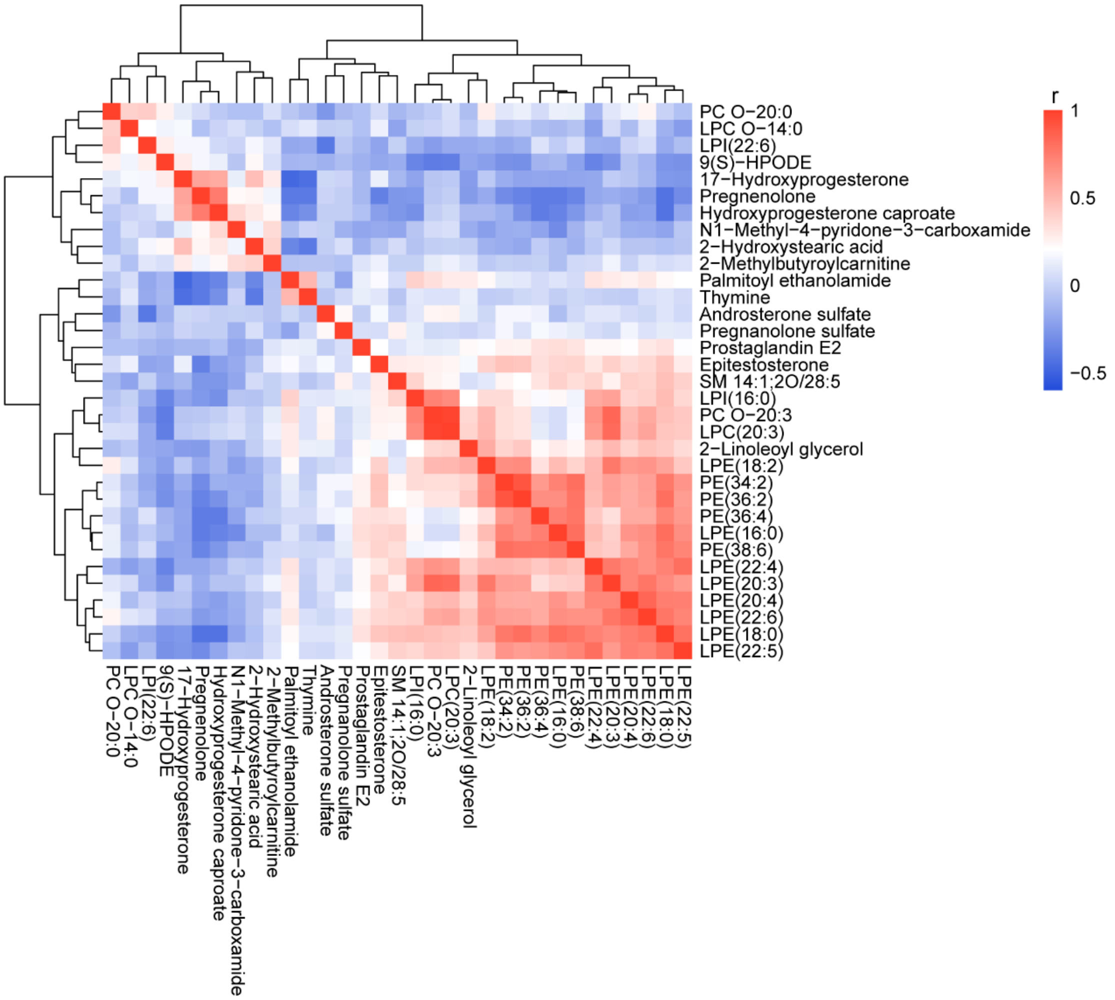

Figure S2. Heatmap of correlations between differential metabolites.

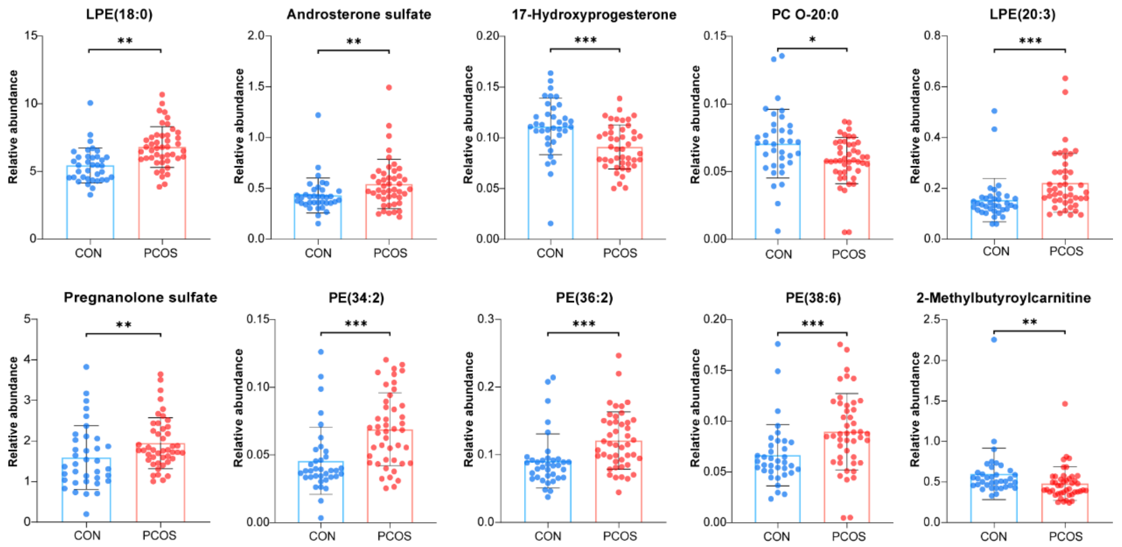

Figure S3. Histogram of the top 10 metabolites according to the ranking of classification importance. The PCOS group is displayed in red, and the control group is displayed in blue. The y-axis represents the integrated data of peak area normalized by TIC from LC-MS. The p values were calculated by the two-sided unpaired Wilcox test. * p <0.05, ** p <0.01, *** p <0.001.

Supplementary Tables

ID	Metabolities	HMDB ID	RT [min]	m/z	Adduct Type	Formula	Relative abundance		log2FC(PCOS/CON)	*P* value	-log10(*P* value)
							CON	PCOS			
RPP1434	2-Linoleoyl glycerol	HMDB0245187	15.86	377.26573	[M+Na]+	C21H38O4	0.1808	0.2301	0.35	2.11E-03	2.68
HIP0796	2-Methylbutyroylcarnitine	HMDB00378	5.36	246.16983	[M+H]+	C12H23NO4	0.5998	0.4786	-0.33	2.49E-03	2.60
RPN1155	2-Hydroxystearic acid	HMDB62549	16.68	299.25817	[M-H]-	C18H36O3	2.3709	1.9296	-0.30	2.01E-02	1.70
RPN0548	9(S)-HPODE	HMDB06940	13.33	311.22166	[M-H]-	C18H32O4	1.7428	1.4154	-0.30	2.06E-02	1.69
HIN0279	Pregnanolone sulfate	HMDB0240590	0.78	397.20496	[M-H]-	C21H34O5S	1.5937	1.9453	0.29	9.89E-03	2.00
RPP0519	Epitestosterone	HMDB00628	11.09	289.21579	[M+H]+	C19H28O2	0.1276	0.1607	0.33	6.00E-03	2.22
RPN0265	Androsterone sulfate	HMDB02759	8.75	369.17264	[M-H]-	C19H30O5S	0.4302	0.5426	0.33	8.33E-03	2.08
RPP0464	17-Hydroxyprogesterone	HMDB00374	10.58	331.22625	[M+H]+	C21H30O3	0.1111	0.0909	-0.29	1.14E-04	3.94
RPP0715	Hydroxyprogesterone caproate	NA	12.69	429.29947	[M+H]+	C27H40O4	0.0547	0.0449	-0.29	2.15E-02	1.67
RPP0580	Pregnenolone	HMDB00253	11.74	317.24702	[M+H]+	C21H32O2	4.0233	3.0824	-0.38	4.74E-03	2.32
HIN0424	Prostaglandin E2	HMDB01220	1.31	351.21801	[M-H]-	C20H32O5	0.0346	0.0529	0.61	2.34E-02	1.63
HIP0554	SM 14:1;2O/28:5	NA	4.06	805.62104	[M+H]+	C47H85N2O6P	0.0233	0.0329	0.50	4.57E-03	2.34

RPP0837	LPE(22:6)	HMDB11526	13.46	526.2923	[M+H]+	C27H44NO7P	1.0584	1.4099	0.41	5.24E-04	3.28
RPP0851	LPE(18:2)	HMDB11507	13.52	478.29232	[M+H]+	C23H44NO7P	2.2671	2.7467	0.28	1.55E-03	2.81
RPP1015	LPE(20:3)	HMDB11484	14	504.30801	[M+H]+	C25H46NO7P	0.1536	0.2216	0.53	1.89E-04	3.72
RPP0850	LPE(20:4)	HMDB11517	13.52	502.29235	[M+H]+	C25H44NO7P	1.5911	1.9625	0.30	9.30E-04	3.03
RPN0761	LPE(22:5)	HMDB11524	14.16	526.29192	[M-H]-	C27H46NO7P	0.1970	0.2673	0.44	6.57E-04	3.18
RPN0735	LPE(16:0)	NA	14.05	452.27632	[M-H]-	C21H44NO7P	4.1462	5.0297	0.28	2.94E-04	3.53
RPN0984	LPE(18:0)	HMDB11129	15.39	480.30755	[M-H]-	C23H48NO7P	5.4419	6.8062	0.32	1.23E-05	4.91
RPN0850	LPE(22:4)	HMDB11523	14.51	528.30753	[M-H]-	C27H48NO7P	0.2393	0.3052	0.35	2.05E-04	3.69
RPN0853	LPI(22:6)	NA	14.54	643.28658	[M-H]-	C31H49O12P	0.2423	0.1996	-0.28	8.99E-04	3.05
RPN1038	LPI(16:0)	HMDB0061695	15.66	571.28682	[M-H]-	C25H49O12P	0.5603	0.6938	0.31	2.59E-02	1.59
RPP1019	LPC(20:3)	HMDB10394	14.04	546.3548	[M+H]+	C28H52NO7P	14.9060	19.0869	0.36	9.34E-03	2.03
RPP0769	LPC O-14:0	NA	13.07	454.32876	[M+H]+	C22H48NO6P	0.1119	0.0778	-0.53	1.27E-02	1.90
RPP0943	PC O-20:3	NA	13.82	546.3548	[M+H]+	C28H52NO7P	1.7293	2.3130	0.42	6.78E-03	2.17
RPP1539	PC O-20:0	NA	16.43	552.40189	[M+H]+	C28H58NO7P	0.0707	0.0582	-0.28	1.02E-02	1.99
HIN0645	PE(36:4)	HMDB09385	3.33	738.50782	[M-H]-	C41H74NO8P	0.0420	0.0543	0.37	2.04E-03	2.69
HIP0502	PE(34:2)	NA	3.5	716.52198	[M+H]+	C39H74NO8P	0.0455	0.0689	0.60	2.77E-05	4.56
HIP0498	PE(36:2)	HMDB09059	3.46	744.55314	[M+H]+	C41H78NO8P	0.0907	0.1210	0.42	8.48E-05	4.07
HIP0493	PE(38:6)	HMDB0009133	3.39	764.52235	[M+H]+	C43H74NO8P	0.0663	0.0897	0.43	4.33E-04	3.36

HIP0346	N1-Methyl-4-pyridone-3-carboxamide	HMDB04194	1.17	153.0658	[M+H]+	C7H8N2O2	0.2686	0.2037	-0.40	6.54E-03	2.18
HIP0386	Thymine	HMDB00262	1.48	127.05025	[M+H]+	C5H6N2O2	0.0206	0.0256	0.31	1.86E-02	1.73
RPP1393	Palmitoyl ethanolamide	HMDB02100	15.63	300.28942	[M+H]+	C18H37NO2	0.0889	0.1168	0.39	1.54E-02	1.81

Table S1. Differential metabolites in FF.
